# What Constitutes Patient-Centered Care in Home Care? A Descriptive Study of Home Health Nurses' Attitudes, Knowledge, and Skills

**DOI:** 10.1097/NHH.0000000000001124

**Published:** 2022-11-04

**Authors:** Mary Curry Narayan

**Affiliations:** **Mary Curry Narayan, PhD, RN, CNS, HHCNS-BC, CTN-A**, is Home Health Clinical Nurse Specialist, Narayan Associates, Vienna, Virginia.

## Abstract

In their seminal paper outlining the proposed *Future of Home Care*, Landers et al. (2016) stated that “patient-centered care” (PCC) is one of the “pillars” of home care. They then asked the question, what is PCC in home care and how is it measured? A qualitative descriptive study explored the answer to this question. In-depth interviews were conducted with 20 home health nurses to identify how they incorporated patient-centered and culture-sensitive care (CSC) into their assessment and care planning practices. The data were categorized into attitudes, knowledge, and skills (including relationship-building, assessment, and care planning skills) associated with patient-centered/culture-sensitive care. The home health nurses had developed multiple strategies for delivering PCC, despite a lack of education in *how* to provide this care. They primarily learned their techniques through their caring for patients as unique, highly valued persons and their ability to form warm caring relationships with their patients. Together they painted a portrait of the attitudes, knowledge, and skills needed for PCC and CSC. PCC and CSC are mutually reinforcing concepts essential to the high-quality, equitable care needed to mitigate healthcare disparities prevalent in home healthcare. A teaching resource for incorporating PCC/CSC into home health clinician practice was derived from the data.

**Figure:**
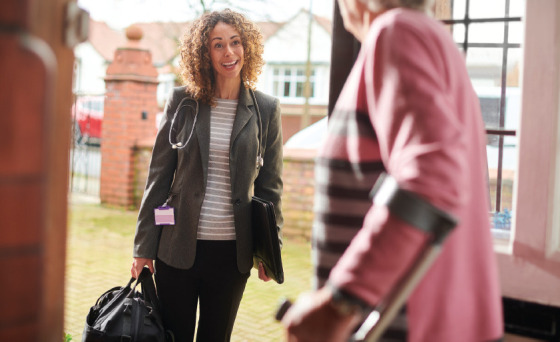
No caption available.

*The Future of Home Health Care* states that one of the “pillars” of home healthcare should be “patient/person-centered care.” They define patient-centered care using the definition in *Crossing the Quality Chasm*: “Patient-centered care (PCC) is providing care that is respectful of and responsive to individual patient preferences, needs, and values, and ensuring that patient values guide all clinical decisions” (Institute of Medicine [IOM], 2001, p. 3; Landers et al., 2016, p. 272). Yet, the *Future of Home Health Care* authors ask, exactly what does PCC look like in home healthcare?

The PCC definition above is an equally good definition for culture-sensitive care (CSC). Indeed, previous research indicates that, at the clinical level, PCC and CSC use the same principles and strategies to help patients achieve optimal health outcomes (Darnell & Hickson, 2015; Lor et al., 2016; Narayan & Mallinson, 2022a; Saha et al., 2008). PCC must be culture-sensitive to be truly patient-centered, and CSC must be patient-centered to be culture-sensitive. Beach et al. (2006) suggest that patient-centered/culture-sensitive care is respectful, individualized care, in which the clinician listens carefully to the patient's values, goals, and preferences; then the clinician engages with the patient as an equal partner in planning care, with the goal of achieving high-quality, equitable care. Therefore, the term “PCC” assumes the care is “culture-sensitive” (Sidebar).

Patient assessment and care planning are crucial for home healthcare. PCC requires an assessment of the factors that affect the patient's health and well-being; and requires nurses to collaborate with patients to develop a mutually agreeable care plan that promotes patient health and well-being. (Well-being includes the emotional, social, and spiritual factors that affect the patient's home healthcare experience.) Because patient assessment and care planning are the basis for home health practice, home health clinicians should provide patient-centered assessments and care planning, but *how* do they do this?

## Literature Review

A medical librarian-assisted search, using various other terms for PCC and CSC, found no published research reports in the nursing or home health literature describing how home health nurses (HHNs) incorporate PCC into their assessment and care planning processes. Broadening the search, we found that even though the term “patient-centered care” is ubiquitous in the nursing literature, there were no studies in the home health literature that used this term. However, by expanding the term to “person” and “client,” and using the British spelling “centred,” six home health studies were found (Brown et al., 2006; Leine et al., 2017; Róin, 2018; Schoot et al., 2005a, 2005b, 2006). All were conducted outside the United States and all, but one, had very small sample sizes (*N* < 10), and generally found nurses had not mastered the communication and partnership skills nor the ability to balance their nursing responsibilities with a patient-centered approach to care.

A search for studies addressing CSC in home health nursing resulted in four studies (Bjarnadottir et al., 2016; Debesay et al., 2014; DiCicco-Bloom & Cohen, 2003; Schim et al., 2006). These few studies suggest that nurses tend to have limited awareness and knowledge about the potential impact of culture on patients' healthcare experiences and that nurses tend to see their patient's culture and lifeways through a judgmental and ethnocentric lens. Other researchers (Debiasi & Selleck, 2017; Delgado et al., 2013; Hart & Mareno, 2014, 2016) found that even when nurses were aware of the importance of culture to healthcare, they did not know how to translate their knowledge into CSC practices.

## Purpose of Study

In *The Future of Home Health Care* report, the authors posed the question: “What constitutes person-centered home health care and how [is it] defined and measured in home healthcare?” (Landers et al., 2016, p. 272). Presuming that PCC is inherently CSC, the purpose of this study was to explore HHNs' attitudes, knowledge, and skills *consistent* with patient-centered/culture-sensitive assessments and care planning.

## Methodology

This study was a part of a larger qualitative multi-method study about patient-centered/culture-sensitive care in home health nursing (Narayan & Mallinson, 2022a, 2022b). After receiving IRB approval, 20 HHNs were recruited through local and national home health agencies, national nursing organizations, a social media group for HHNs, and snowball sampling. Eligible participants worked for Medicare-certified skilled agencies, had at least 1 year of home health experience, and performed patient assessments and care planning or educated or supervised those who did. The participants were primarily White females (1 Black, 1 male). Almost all were currently working for Medicare-certified, skilled home health agencies located in 11 states throughout the United States. Their average age was 52.5 years (range: 25 to 68 years). Educational preparation included ADN (4), BSN (11), and MSN/MS (5) degrees. Most worked as case-managing clinicians; some had supervisory or educational roles on a part- or full-time basis.

The HHNs participated in approximately 1-hour, audio-recorded, individual, in-depth interviews, which occurred between January and July 2020. A semistructured interview guide with open-ended questions was used to determine how HHNs acquired patient-centered/culture-sensitive knowledge and skills and how they incorporated these skills into their assessment and care planning activities. This article addresses the patient-centered/culture-sensitive attitudes, knowledge, and skills of HHNs.

In this analysis, HHNs' attitudes, knowledge, and skills were compared with the PCC and “caring” nursing literature (Leininger & McFarland, 2006; McCormack & McCance, 2017; Ray, 2016; Watson, 2008) and the transcultural nursing literature (Andrews et al., 2020; Douglas et al., 2014; Giger & Haddad, 2020; McFarland & Wehbe-Alamah, 2018; Purnell & Fenkl, 2021; Ray, 2016). I also compared them with the attitudes/knowledge/skills of nurses certified by the Transcultural Nursing Society in providing CSC. I extracted the HHNs' quotes that reflected patient-centered/culture-sensitive attitudes/knowledge/skills.

The skills category was subdivided into three types of skills. Tables 1-5 show examples of the data (quotes) for each of the five categories: attitudes, knowledge, relationship-building skills, assessment skills, and care-planning skills. Each of the categories was analyzed for themes and subthemes shown in Tables 1-5. Edits were made to the quotes to shorten them, provide essential context, and render them grammatically easier to read, while maintaining the meaning of the quotes.

**Table 1.** Attitudes & Values Consistent with PCC/CSC

Theme	Subthemes	Representative Quotes
Caring	Heartfelt caring for each patient	So I tell my patients, “I'm giving you all that I can give you when I'm here with you, and I'm giving it to you from the heart and I'm treating you like you're my mother, my grandpa.” P15 People understand genuineness. When you are genuinely caring and concerned about them, even across cultures, they respond to that and understand that. So you need to be genuine and authentic in your caring. P20
	Attentiveness, presence to patient	Your work is not just changing the dressing. It is about getting to know about the person inside the patient. You have to want to promote their well-being, not just heal their wound. P3 You need to be more sensitive to what's going on with your patients, hearing what the patient has to say. Listening is a huge part of “being present.” P14
	Empathy, understanding of patient as unique person	Get out of your head, get into theirs. Put yourself inside them. What do they want, what do they need? P5 You have to be motivated to want to understand the person, especially patients whose lifestyles are different from your own. P12 To be patient-centered, the nurse needs empathy, compassion, and curiosity. P20
	Acceptance, nonjudgment	To have a good relationship with patients, nurses need kindness, kindness, kindness. Empathy. Compassion. Understanding. Being nonjudgmental. P15 I guess it just comes down to the ability to be nonjudgmental, and to look at each patient as an individual. P16
	Spiritual/humanistic commitment, deep desire to help people	I get to use my skills to hopefully improve my patient's lives a little bit, to make a difference in the world. All of us can be so profoundly instrumental in improving the health and wellness of our society. P13 I love what I do. I care about what I do. P15
Flexibility, adaptability		I think flexibility is the key when you're talking about patient-centered care. You've got to be flexible and adapt your style to the patient's needs and wants. P13
Commitment to ongoing learning		You have to be willing to learn and willing to do some research in other cultures. P10 You need to be willing to learn from your patients, to ask questions about differences, to respect what they believe, and to understand it. P19

*Note*. P# = Participant's identification number

**Table 2.** Knowledge Helpful to Providing PCC/CSC

Theme	Subthemes	Representative Quotes
What to Learn	Self-knowledge as “cultural being” with biases	Of course, I have unconscious biases, but I try to be professional and overcome them. You look at yourself and remove your barriers. Look at what you do and look at how you could do it differently. P13 I think it helps to know one's own cultural norms, what impacts my own health and my own wellness. So just understanding what is important in your life that would impact your own health goals and getting better. It helps you understand what is important to your patients. P19
	Knows about types of cultural/lifestyle variations	I make a lot of eye contact with my patients. But in certain cultures, that's offensive. Another thing is touching or exposing patients. It's always good to know the norms of the patient's culture. P1
	Knows each patient is an individual, not a stereotype.	I think culturally competent means looking at every person as an individual human being. P9 Every culture is different. Every person within a culture is unique. They have their own unique belief system and their own experiences. P14
How to learn	Knows how to get cultural info from resources	If I get somebody who is obviously from another culture, I'll look up the culture a little bit. “I google it.”
	Knows how to ask patients sensitive questions	I say, “Please tell me if I'm doing anything that makes you uncomfortable. I don't know about your culture, and I don't want to offend you.” P9 When I don't understand something about their culture, I'll ask them and just tell them “I want to understand and I want to respect what your wants, wishes and desires are.” P15 You need to know how to listen, and you need to know how to probe and ask the questions in a sensitive and thoughtful way. P20
	Knows how to think critically & creatively	I am able to look back on things that didn't go well and to think about what could have been done differently. P11

**Table 3.** Relationship-Building Skills that Facilitate PCC/CSC

Theme	Subthemes	Representative Quotes
Start off on the right foot	Be courteous cheerful, positive, & appear relaxed.	You have to have a happy, cheery tone of voice. P4 If we have a more easy-going attitude as well as positivity, then our patients will be more prone to open up to us and allow us to help them. P18
	Appear professional, knowledgeable, confident.	It's important to present that confident, professional persona right up front. P9 Be professional and courteous. Show up on time, be professionally dressed, all that kind of thing. P10 Nurses need to be knowledgeable—about diseases, latest best practices, cultures—so they can be confident. P16
	Socialize before business	You've got to build rapport with them first. P5 I just think that the relationship is absolutely the most important thing. Socialize a little bit, till they feel more comfortable. P8
	Include family/caregivers	Talk directly to the patient and the family. Don't talk *at* them but include them in the conversations and ask them their opinions. P6
Build a warm trusting relationship	Build trust	It is a relationship. You treat them as a person, not a task. That's how you get therapeutic communication and a therapeutic relationship. P5 They need to see that you are there for them. So I think, building the trust, letting them see that you are on their side, and that you really are there to help them. P9
	Listen carefully to show caring	I tell my patients to “tell me your story,” asking them to tell me about what happened to them related to their illness or injury. P1 Really work on active listening. I know it's hard. I know we're in a hurry. P12 Take time to listen, and to truly understand your patients. P17
	Share power with the patient	You need to respect that you're in someone else's home. You're not in charge here. The patient is in charge and the only way you're ever going accomplish what you want is if you let them be in charge. P1 It's their territory, their home, their turf. You're a guest. You can't come in and say, “This is what we're doing, this is how we're doing it, and this is going to be the way it is.” P5 When patients have more active control over their care, rather than just being the one that doesn't have a say, feeling like someone just comes in, does what they do, and leaves, then they feel much more satisfied and much more comfortable and at peace. P20
	Assure good communication	I speak some Spanish, but if they're asking me questions and I'm not understanding, then I get the language line involved, and I have that on speed dial on my work cell phone. P9 Listen to your patient, and then communicate within the patients' health literacy and cultural beliefs. P16
	Humanize yourself	Be a person. I think it helps them to relate to you. Pull out something you share with the patient, like a hobby or grandchildren. P1 When assuring that patients are taking the right medications, “I tell them about a medication error that I made on myself, and I had to tell the doctor. And then we laugh together about how I could have made such a silly error. And then they listen when I recommend using a medication box.” P4

**Table 4.** Assessment Skills that Demonstrate PCC and CSC

Theme	Subthemes	Representative Quotes
Content	Conduct a patient-centered (individualized) assessment	You have to know your patient; you have to know what's going on with the patient. I think the assessment, and patient-centered care are all wrapped up together, because you can't understand your patients without a thorough assessment. P2 You can't do the same assessment for every single person. You should have a baseline, but whatever comes up, you need to focus on that. P10 I identify what's important to the person because if you listen, they'll usually tell you what would be most helpful to them. And so that's usually where I start. It's whatever is most on their mind, most problematic, what they're most scared about or concerned about. P12
	Conduct a culture-sensitive assessment	I want to make sure I am not judgmental, instead I am just curious. I am attentive to their milieu, and then I start to ask questions. I want to know what their belief system is, their cultural interpretation of things, and how they feel about what's going on. I also ask, “What has your experience been with healthcare providers? What's been good, what's been bad?” So that we can make sure that we're starting where they are at when we give them information, so they can understand and can make it their own and buy into it. P14
	Conduct a holistic assessment	You have to look at the whole person, not just the disease. We have to pay attention to the whole human being. P9 Your assessment is obviously going to include the physical assessment piece, but it also includes an assessment of how they really are doing. How are they functioning at home? How does their environment impact them? What kind of needs do they have beyond the obvious healthcare needs? Paying attention to just really a lot of things. P16
	Assess psychosocial factors	You need to assess environment and social issues, which are frequently more important than their physical assessment. P3 The family dynamics, environment, finances, everything plays a part in trying to get them managed at home and you have to deal with all of those things. P7 I try to put the whole picture together by using the OASIS as a starting point. So as I'm going through the OASIS, I'm doing that whole comprehensive psychosocial assessment. P13
	Assess learning needs	I find out where they are at in their trajectory of owning their health and wellness and management of their disease, rather than their disease managing them. I ask, “How confident are you that you can manage whatever their primary issue is. I ask them and the family that.” I ask, “Does this disease control you, or do you control the disease?” Opening the door to the conversation. P13 Assess what they know and then work from there. Don't ever assume that the patient knows. Ask what has the doctor told you? What did you learn in the hospital? So that we can kind of take it from there. Not reinvent the wheel and not confuse them with giving different information. P14
Processes	Converse, don't interrogate	So a lot of times when we're doing an OASIS, we're able to really dig into their health history and really help them understand things. I use the OASIS as a springboard for deeper questions... So we just kind of get into more of a conversation. P18
	Follow-up on patient cues	To do a good assessment, you have to combine your critical thinking with empathy. You have to pick up on the patient's cues. P11 I usually take cues from the patients. So whatever issue they identify, I just kind of listen and further develop that until it's kind of done. Because I sometimes think if you don't listen to them, they can't hear you at all. P12

**Table 5.** PCC/CSC Care Planning Skills

Theme	Subthemes	Representative Quotes
Identify patient's goals		“What do you think we need to work on? This is what I think? What do you think? What are your goals? What's your most important goal?” P4 What does the patient expect and hope for related to their health problems? What do they want? What are their goals? What are the barriers that they may face? P16 “I ask them ‘What do you want to get out of our services? What are your health goals? What do you want to see happen in X amount of time?’ A lot of patients have a real hard time coming up with their goals. Often I get to help them through thinking about what their goals might be. Then we have a discussion about what their goals are and what my goals are. You address the goals each time you go to see the patients. What I typically say when I go back to the patient, ‘In terms of reaching your goals, what went well this week? What could have gone better, or what didn't go so well?” P17
Engage patient & caregiver in planning		Tell the patient that home care is a team approach and that they're part of that care team and that the goals we're working on should be ones the patient is invested in. Any goals you come up with, you have to assure the patient agrees because if the patient doesn't see the value in the goal, you won't get the outcome you want. P11 After establishing the goals, say, “Let's make a plan together to reach these goals.” P14
Tailor the care plan to patient		Really individualized care is the hallmark of what we need to do. P8 Think of the person as an individual, their background, their culture, who they are, listen to them and develop a plan of care based on all that information. P10 The computerized diagnosis-based care plans are helpful, but they need to be individualized to each patient. P15 What's going to improve their life given their healthcare situation? Develop that plan of care and incorporate their desires. P16

## Results

Patient-centered/culture-sensitive attitudes, knowledge, and skills occurred along a continuum from modest to robust. Almost all the participants said they had not received any education about PCC or CSC or the education they received was not adequate for the diversity challenges they encountered in their work. Although some nurses said they were unfamiliar with the terms “patient-centered care” or “culturally competent care,” all exhibited some culture-sensitivity and patient-centeredness. They seemed to develop these qualities by combining their “caring” for each patient with reflection on their experiences, gradually learning how to be more effective through patient-centered/culture-sensitive attitudes, knowledge, and skills.

### Attitudes & Values (Table 1)

All participants described caring about their patients. “Caring” was the source and primary theme for most of their other patient-centered/culture-sensitive attitudes (subthemes of caring). For example, participant 7 (P7) revealed, “*I always think about my patients as if they were my grandparents. How do I want them taken care of? That's what I want for my patients*.” They described “caring” as seeing the patient as a valuable unique person, caring about who the patient is as a person, and caring about what the patient cares about.

Caring was described as empathy, compassion, nonjudgment, and kindness, which depended on “getting inside the patient's perspective” (P5) about their health issues. As P20 said, “*When my cultural beliefs butt up against a patient's cultural beliefs, it reminds me that I'm thinking through my own cultural lens and not theirs*.” Several HHNs discussed the importance of “meeting patients where they are” physically, cognitively, and emotionally. Some HHNs stressed the importance of being present to the patient through careful attentive listening, until the nurse understood what the patient was experiencing, thinking, and feeling, and then “being non-judgmental, open and receptive” (P10).

A few participants discussed the need to be flexible and adaptable so they could tailor their assessments and care planning to each patient. Others discussed the need to have an ongoing commitment to learning (a willingness to give time to learning about best practices and cultures), so each patient received quality care. For instance, P9 stated, “*The diversity of my patients keeps me looking things up and makes me continue to do research into different things*.” Other attitudes included optimism (conveying optimism to the patient about their ability to achieve good outcomes), commitment to the patient (willingness to work extra time to provide patient-centered/culture-sensitive care), and resilience (belief that the nurse can overcome challenges to PCC care through critical, creative thinking, and adaptability).

### Knowledge (Table 2)

The themes for knowledge fell into two categories: *what* the participants felt they needed to know to deliver patient-centered/culture-sensitive care and *how* they learned what they learned. In the what-to-know category, several HHNs commented on the value of self-knowledge, knowing themselves as “cultural beings” with their own positive and negative biases and their own preferences for certain beliefs, values, aspirations, behaviors, and practices. They felt that self-knowledge helped them to manage their biases and helped them to be more accepting of patients' differences.

**Figure 1.**
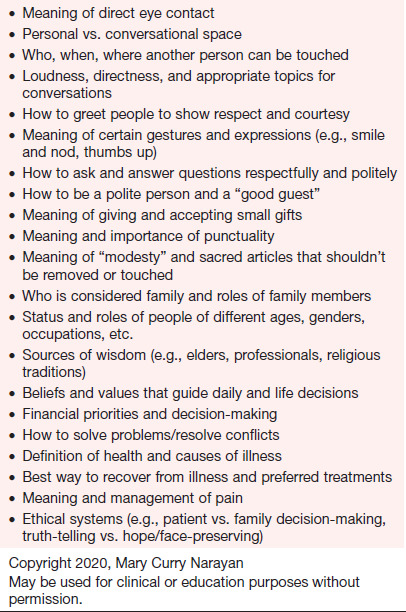
Ways cultural groups may differ from American and western medical norms

Another crucial area was general knowledge about the ways cultural groups could differ from expected American and western medicine norms (Figure 1). Several participants said nurses need to be familiar with the cultural and lifestyle norms of the populations they serve to avoid misunderstandings and to avoid inadvertently being offensive. For instance, P20 noted that not understanding the communication norms of one group left her feeling intimidated. “*The way they talk in certain cultures came across as aggressive or angry. It almost sounds like they are yelling at you. But if you know that's just the way they communicate – very loudly – then you're no longer threatened*.” Several HHNs commented on needing to know each patient as a unique person, instead of a stereotype of any of the groups they may be part of. P5 said, “*Everybody is different. Everybody is diverse. I just try to think of each person as an individual. What is this person's story? What are this person's beliefs, ways of doing things?*”

When asked *how* they learned their patient-centered/culture-sensitive knowledge, there were three types of responses. First, they learned by consulting websites, books, journals, or cultural experts. For instance, P14 described how nurses at her agency learned about cultural norms. “*Some of the nurses took it upon themselves to really study and learn and became knowledgeable about the norms of a population we were serving, and then they shared their knowledge with the other nurses at case conferences*.” Participants said it was important to learn how to ask cultural and lifestyle questions in a sensitive way. P5 suggested asking, “*Is there anything I should or shouldn't do to honor your culture [or lifestyle]?*” P10 said he realized that it is culture-sensitive *to* ask culture-sensitive questions, saying, “*I think for the most part, patients appreciate those kinds of questions. It lets them know you're thinking about them as an individual*.”

Many nurses reported they learned their patient-centered/culture-sensitive skills through a trial-and-error process of thinking reflectively on patient experiences with their critical and creative thinking skills to figure out effective ways to be more patient-centered and culture-sensitive. For example, P9 reported how she used this process to enhance her skills. “*At first, I went in to fix things, and then after a few times I thought, I don't know anything about this culture. Why am I trying to fix what I don't understand? And it's at that point I started asking questions. If you don't know why the patient is doing something, or how it is related to their culture or lifestyle, the right thing to do is to ask people questions*.”

### Relationship-Building Skills (Table 3)

Almost all the participants felt the most important step for conducting a good assessment and creating an effective Plan of Care was to build a warm, trusting relationship with the patient and family/caregivers. They spoke about making a good impression from the first phone call and visit, being courteous, cheerful, positive, and relaxed—a pleasant person who “has time” for the patient as a person. They also felt nurses should project a knowledgeable professional demeanor that inspires confidence. Several HHNs talked about the importance of social small talk before getting to the business of the visit. For instance, P7 said, “*I don't really rush into anything with them. I sit there and I do small talk with them for a while whether it be about the picture of their grandchild or their collection of the dolls. I establish a relationship with them before I start getting forms signed and doing the assessment*.”

The participants had several recommendations for deepening the warm trusting relationship. P2 reported, “*You have to build that trust between you and the patient. That, of course, takes time. People don't trust someone right away. It's just listening, making the patient feel they're very important to you*.” Thus, continuity of care (one primary nurse for each patient) and listening carefully to the patient help show patients the nurse truly cares about them, which builds the relationship. P20 said, “*Listen carefully, so the patient feels heard and respected. When they feel heard and respected it gives them more comfort in sharing their needs and desires than they otherwise would. And I think it gives them more power to be involved in planning their care*.” Sharing power with the patient not only builds the relationship, but it also is the only way to develop a truly effective care plan. As P15 explained, “*If you don't respect the patient's concerns and preferences, it affects how much you are going to be able to accomplish because they are going to put up a wall between you and them. If you are not willing to work with them, nine times out of ten they're not going to work with you*.”

A few nurses mentioned the need to assure good communication with the patient, which requires the nurse to meet patients within their language (through medical interpreters) and their health literacy (using terminology and sentences that mirror the patient's health literacy). Another strategy for building the relationship that several nurses mentioned was sharing a little bit of who they are as persons, which humanizes themselves, demonstrating the nurse is like the patient. P5 explained, “*Share a little information about yourself to let them see you are a person, and not just the nurse. I think that it helps build the rapport and makes them more comfortable with you. If you want to have a good therapeutic relationship, they've got to like you. So, you open up. You show them that you're human*.”

### Assessment Skills (Table 4)

Participants felt that assessments should be holistic, individualized (patient-centered), and/or culture-sensitive. They described holistic assessment as assessing factors that affect the patient's ability to heal and recover, like psychosocial, cultural, spiritual, and financial factors. For example, P7 said, “*Your assessment should include a lot of factors because lots of factors affect their ability to reach healthcare goals*.” P12 stated, “*Patients live and get better holistically, so they need holistic assessments so we can provide holistic care*.”

A few HHNs discussed how they individualized their assessments to their patients' unique circumstances, which is a hallmark of PCC. P5 said, “*You have to meet them where they're at. So, I tailor my assessment to how the person presents. It just depends on what they say and what they tell me and then I tailor what I do and say to them, from the type of language I use, to the way I phrase questions, to the order in which I assess things*.” To make their assessments culture-sensitive, a few participants described how they questioned patients about their cultural norms. P8 explained, “*If there is something I don't understand, I say, ‘This is something I'm not familiar with, but I want to make sure that we understand what you'd like us to do. Can you explain to me a little bit about how your culture influences your healthcare? I want to make sure we're respecting your wishes*.”

Participants tended to discuss two assessment areas in particular: psychosocial assessment and assessment of learning needs, which are more important to home healthcare outcomes than in many other nursing settings. P8 discussed the importance of the psychosocial assessment. “*Even though it's something that may not relate to the physical health of the patient, their mental health is just as important and the things going on around the patient affect them. There are social issues in the home that are a bigger need at the moment. So, we need to take care of all those things*.” Other nurses focused on the learning needs assessment, such as health literacy level and what the patient already knew about their condition and its management. For instance, P10 reported, “*If their problem is diabetes, I say, ‘Tell me what you know about diabetes. What has your doctor told you your blood sugar range should be? Tell me how you control your blood sugar.’ So, I ask questions, almost like doing a Teach-Back thing*.” P13 described how she used motivational interviewing and the Importance/Confidence ruler to determine the patient's ability to manage their health problems.

Finally, a few participants discussed techniques to assure a patient-centered assessment. P7 described how she made her Outcome and Assessment Information Set (OASIS) assessment a conversation. “*I know the OASIS well enough to get the answers to those questions in a conversation. There are very few things I actually point-blank ask; it's all in conversation*.” Another technique was to pay attention to patient cues. P16 commented on how easy it is for nurses to *not* hear when patients cue us to their needs and concerns, even though hearing those cues is a patient-centered requirement. “*I think nurses get so task-oriented, and we've got our computers in front of us doing the OASIS, that when patients drop cues about other needs, we don't' even hear them, so we don't follow up*.” P10 emphasized the importance of hearing those cues. “*I would say an effective assessment tool is being an active listener, hearing and responding to patient cues. Just follow-up on cues, diving deeper into wherever the patient leads you*.”

### Care Planning Skills (Table 5)

Several participants outlined a PCC planning process. First step is to identify the patients' health/well-being goals (expected outcomes). As P5 said, “*A good care plan starts with asking the patient, ‘What are your goals? Where would you like to see your health in the next two months? What do you want to be able to do?’ Then you figure out how we are going to get them there*.” P12 described how she combined the patient's goals with the medical goals for a particular problem, explaining, “*Although the patient's goals seem to differ from typical medical goals, usually their goals and your goals overlap. They say it in a personal way, so you can incorporate your goals into their framework*.” P17 described how she helps the patient come up with their goals and then how she follows up with the patients' progress toward their goals each visit.

After identifying the patients' goals, a few participants described how they engaged patients in care planning, telling them that they are a crucial member in the care team and seeking their opinions on how to achieve their goals. They suggested that if patients are part of the care planning process, they will be more motivated and adherent to the plan. For instance, P6 explained, “*You've got to include the patient in planning the care plan. Make sure the patient helps develop the plan. If they feel engaged, then they're more likely to follow the plan*.”

The final step in the care planning process is to assure the Plan of Care is tailored to the patient's lifestyle and preferences, instead of telling the patient to adhere to the computer-generated, diagnosis-based care plan. As P5 remarked, “*This patient is an individual. They have individual needs. I cannot put them in a mold. I cannot use a cookie cutter on them*.” P14 advised, “*You've got to build the Plan of Care around what's going to work for the patient, or you end up with a non-compliant patient*.”

## Discussion

**Figure 2.**
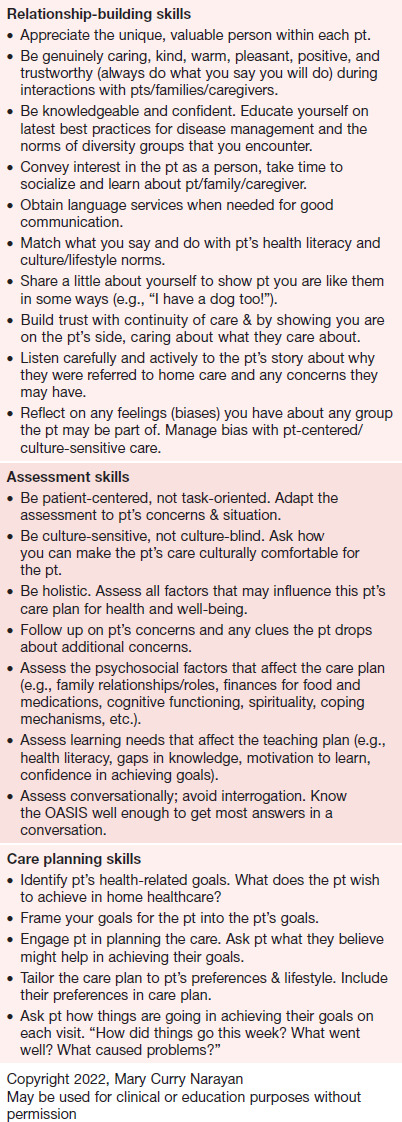
How to provide patient-centered assessments and care planning

The results of this study—the lists of patient-centered attitudes, knowledge, and skills that HHNs incorporate into their daily assessment and care planning activities—begin to answer the *Future of Home Care* authors' (2016) question about what PCC is in home care. *Together*, these HHNs painted a portrait of what PCC can be in home health nursing care. The results provide an initial outline of how to incorporate PCC into the nursing assessment and care planning. Our knowledge about how to incorporate PCC into home healthcare can be developed and refined, however, a resource for beginning to integrate PCC into home health assessment and care planning activities was derived from the data (Figure 2).

**Challenges to PCC**. The methodology of this study—in which I began by identifying HHNs' statements that demonstrate patient-centered attitudes/knowledge/skills—did not tell the whole story of the status of PCC among these participants. Although all HHNs in this sample exhibited at least some of the attitudes/knowledge/skills that a sample of transcultural nurses identified as being part of their daily assessment and care planning practices (Narayan & Mallinson, 2022a), none of the study participants exhibited *all* the attitudes/knowledge/skills noted by transcultural nurses. In fact, the data revealed that some nurses were *not* patient-centered and were culturally *in*sensitive in several ways. These included 1) a lack of knowledge of the principles and strategies of PCC or CSC, 2) biases and stereotypes about some patient populations, 3) authoritarian versus collaborative attitudes about care planning, 4) cutting corners (i.e., neglecting to use patient-centered/culture-sensitive strategies) to promote efficient visits and accommodate productivity requirements, and 5) a task-oriented (care focused on the minimum tasks needed to complete a visit) versus a patient-centered approach to care, among others. Although some nurses were successful at achieving PCC, some were less successful, leaving patients, especially minority patients, at risk for poorer outcomes.

**Importance of PCC**. Patients have a right to high-quality and equitable outcomes. One way to promote high-quality care is through PCC (IOM, 2001) and one way to promote equitable care is through CSC (IOM, 2003). Yet, home healthcare patients of various minority and marginalized groups experience disparate outcomes (Chase et al., 2018; Joynt Maddox et al., 2018; Miner et al., 2020; Narayan & Scafide, 2017; Smith et al., 2021), suggesting they may not be receiving high-quality, equitable care (IOM, 2001, 2003). Yet, according to the *Code of Ethics for Nurses*, “The nurse practices with compassion and respect for the inherent dignity, worth, and personal attributes of every person, without prejudice” (American Nurses Association, 2015, p. 11). PCC outlines how to put this provision into practice. In addition, the Committee on the Future of Nursing 2020-2030 (2021) states that, in the United States, nursing's highest priority is to achieve equitable care for all patients and that one of the ways to achieve this goal is through PCC. By recognizing PCC as a pillar of home care, the *Future of Home Care* authors (Landers et al., 2016) acknowledged and promoted PCC as an essential element of home care practice; it is a “must-do,” not a “nice-to-do,” component of practice.

**Importance of genuine caring and education in PCC skills**. The study indicated that PCC is associated with nurses' genuine caring for each of their patients as deeply valued individuals. This finding is related to a previous grounded theory study, which found that “caring” is the source for HHNs' journey toward learning PCC and CSC (Narayan & Mallinson, 2022b). That study further found that HHNs learn their patient-centered/culture-sensitive skills through an arduous, experiential trial-and-error process motivated by their caring, rather than through preemptive education in nursing school, orientation to home care or in-services. Although genuine caring can lead to patient-centered/culture-sensitive skills, education in how to incorporate patient-centered strategies into daily practice will help HHNs learn these skills more effectively and universally, enabling more home health patients to receive high-quality, equitable care.

**Strengths/Limitations**. Because of the methodology of the study—identifying nurse comments that demonstrate patient-centeredness and culture-sensitivity—no conclusions can be made about the status of PCC in home healthcare. As the interviews elicit self-reported data, social-desirability bias is a risk. With a quarter of the sample having MSN/MS degrees, the participants were more educated than those routinely hired to provide case management and visits to home health patients. This made the sample less representative of the population of HHNs who assess and plan care for patients. However, it was a strength for this study because these nurses tended to be more familiar with the principles and application of patient-centered and culture-sensitive care and had insights about how to put it into practice.

### Implications for Home Healthcare

Although this study focused on HHNs, many of the nurses' strategies may prove helpful to other home care clinicians, especially rehabilitation therapists. Clinicians may wish to use the results of this study to evaluate their own attitudes, knowledge, and skills and to understand their own PCC strengths and weaknesses. To enhance their own relationship-building, assessment, and care planning skills, they could use the handout (Figure 2) to gradually adapt and modify the suggested strategies into their own style.

Home health educators may wish to incorporate a PCC module into their orientation and in-service programs, to explain why PCC is important in preventing disparities, and ways to integrate patient-centered strategies into their practice. Administrators may wish to integrate PCC into their agencies' mission statements, orientation, education, and evaluation programs to improve the quality of care, to prevent disparities within the minority and marginalized patient populations they serve, and to improve their quality indicator scores. Researcher*s* may wish to replicate this study with therapists, or they may wish to broaden the investigation into what other clinical strategies clinicians can use to make their PCC interventions more effective. Researchers may also wish to explore what patient-centered and culture-sensitive care is from patients' perspectives. They may want to investigate the effect of a PCC educational program on selected quality indicators, such as patient satisfaction with care. Researchers could also investigate if the identified patient-centered attitudes/knowledge/skills can be used as an objective PCC evaluation tool to help measure indicators of what PCC is in home healthcare.

## Conclusion

This study identifies the attitudes/knowledge/skills of a sample of HHNs that are consistent with PCC. Together, the HHNs painted a portrait of PCC and made recommendations that can help home health clinicians enhance their ability to deliver it. Paradoxically, it also identified that some nurses have a task-oriented and culture-insensitive approach to care that can affect the quality and the equity of care delivered to home health patients.

If PCC is a pillar of home healthcare, then clinicians need to master its principles and strategies, so they can deliver high-quality equitable care. Evidence shows that healthcare disparities persist in home care and that providing PCC can decrease those disparities. PCC in home healthcare is a moral imperative. Mirroring the patient-centered attitudes/knowledge/skills captured in this study may help home health clinicians enhance their patient-centered skills.

## Sidebar: Notes about Terminology

**Culture**. “Culture” refers to the normative beliefs, values, preferences, lifestyles, etc. of any group that ascribes to a particular identity, including race, religion, gender, age, socioeconomic status, ability/disability, sexual orientation, gender identity, occupation, ideology, and many other ways people with a shared identity differ from majority populations.

**Culture-sensitive care (CSC)**. I use the term “culture-sensitive care” instead of “culturally competent care,” which has some unfortunate connotations: 1) It is perceived by many as knowing a lot about other cultures instead of the ability to respect and adapt to patients' cultures; 2) It is about clinicians' abilities instead of patients' needs, so it is not patient-centered; 3) It lacks the concept of humility needed to deliver care across cultures.

**Patient-centered care (PCC)**. Although many nurses prefer the term “person- or client-centered care,” I use the term “patient-centered” because it connotes the ethical relationship nurses have with patients. Patient-centered care always includes care that is sensitive to the patient's cultural beliefs, values, needs, and preferences.

**Patient-centered/culturally sensitive care**. I use this term to reinforce that patient-centered care is always culture-sensitive and culture-sensitive care is always patient-centered.
